# Echocardiography-Guided Repositioning of a Hemodialysis Catheter in a Patient With Persistent Left Superior Vena Cava: A Case Report

**DOI:** 10.7759/cureus.101971

**Published:** 2026-01-21

**Authors:** Yusuke Nozaki, Junki Kinoshita, Kentaro Miyake

**Affiliations:** 1 Department of Anesthesia, Kasugai Municipal Hospital, Kasugai, JPN

**Keywords:** catheter repositioning, coronary sinus (cs), hemodialysis central venous catheter, persistent left superior vena cava (plsvc), transthoracic echocardiography (tte)

## Abstract

Persistent left superior vena cava (PLSVC) is an uncommon thoracic venous anomaly that is often identified incidentally during central venous access or thoracic imaging. We describe a case in which transthoracic echocardiography (TTE) was used to safely and effectively reposition a hemodialysis catheter within a PLSVC to enable continuous hemodiafiltration (CHDF). A 71-year-old man developed septic shock complicated by acute kidney injury requiring CHDF. Because vascular access options were limited, a dialysis catheter was inserted via the left internal jugular vein. Imaging revealed that the catheter was located within a PLSVC, and TTE demonstrated that the catheter tip was positioned near the coronary sinus, raising concern for potential cardiac perforation. Under real-time TTE guidance at the bedside, the catheter was withdrawn several centimeters to a safer position. CHDF was subsequently performed for nine days without complications, maintaining a stable blood flow rate. TTE is a valuable bedside tool for confirming and adjusting catheter position in a PLSVC, particularly in critically ill patients who are unsuitable for fluoroscopic imaging.

## Introduction

Persistent left superior vena cava (PLSVC) is the most common congenital thoracic venous anomaly. While its exact frequency is unknown due to its often asymptomatic nature, the prevalence is estimated to be between 0.2% and 3% in the general population [1]. In a prospective echocardiographic study of 2,841 normal neonates in Japan, Nagasawa et al. reported an incidence of PLSVC of 0.21% (95% confidence interval 0.042-0.38%), whereas the incidence was more than sevenfold higher among 1,920 patients with congenital heart disease [2]. In most cases, the PLSVC drains into the right atrium via the coronary sinus and is hemodynamically insignificant, rendering it asymptomatic [1]. Consequently, PLSVC is often detected incidentally during procedures such as central venous catheterization or thoracic imaging [1,3]. 

In the most common variant, the PLSVC descends along the left side of the mediastinum, lateral to the aortic arch, and drains into the right atrium through an enlarged coronary sinus, creating a potential pathway for a catheter inserted from the left neck. The junction between the PLSVC and the coronary sinus can be thin-walled and acutely curved; therefore, a stiff catheter tip abutting this segment may increase the risk of perforation and cardiac tamponade. Although a PLSVC can serve as an alternative route for vascular access when conventional central veins are unavailable, determining the optimal catheter tip position remains crucial to avoid complications such as arrhythmia or cardiac perforation [3].

Fluoroscopy has traditionally been used to guide catheter placement in these patients [3], but transferring critically ill patients for imaging carries substantial risk. Therefore, a reliable and non-invasive bedside alternative is desirable. Transthoracic echocardiography (TTE) is non-invasive, portable, and can provide real-time imaging of cardiac structures and adjacent great veins, making it a potential alternative for catheter visualization at the bedside. Here, we report a case in which a hemodialysis catheter was safely repositioned in a patient with a PLSVC using TTE at the bedside. This case highlights the practical value of TTE for ensuring safe catheter positioning in critically ill patients.

This article was previously presented as a meeting abstract at the 2025 Japanese Society of Cardiovascular Anesthesiologists (JSCVA) Annual Scientific Meeting on September 19, 2025.

## Case presentation

A 71-year-old man was brought to the emergency department with an acute disturbance of consciousness. He presented with fever (38.4°C), tachycardia (113 beats/minute), and a Glasgow Coma Scale score of E4V2M4. Blood pressure was 139/70 mmHg, respiratory rate was 23 breaths/minute, and oxygen saturation was 93% on room air. Physical examination revealed pitting edema of the lower extremities and scrotum.

**Figure 1:**
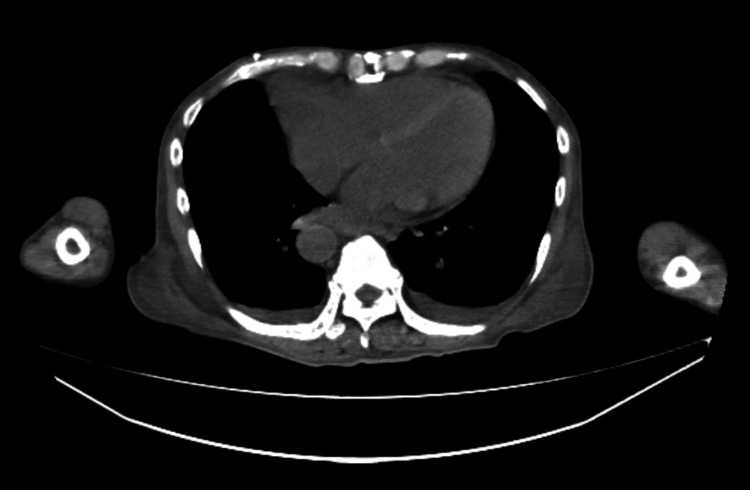
Chest computed tomography showing bilateral pleural effusions and subcutaneous edema

**Figure 2:**
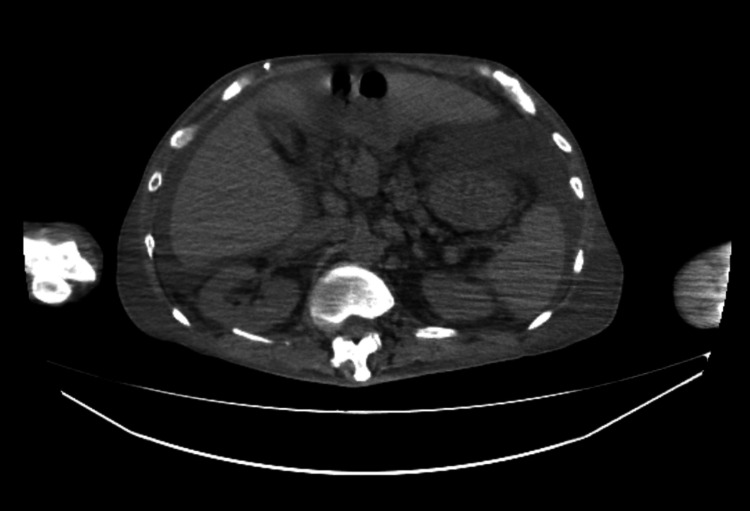
Abdominal computed tomography demonstrating large-volume ascites

Initial laboratory data are summarized in Table 1 and showed elevated inflammatory markers, mild renal dysfunction, thrombocytopenia, and coagulopathy. Chest computed tomography (CT) demonstrated bilateral pleural effusions and marked subcutaneous edema (Figure 1). Abdominal CT from the upper abdomen to the pelvis showed large-volume ascites surrounding the liver and filling the pelvic cavity (Figure 2). Bedside TTE demonstrated preserved left ventricular contractility (Video 1). He was diagnosed with sepsis of unknown origin and treated with piperacillin-tazobactam and continuous norepinephrine infusion.

**Table 1:** Laboratory findings at admission and ICU day 7 Key abnormalities included marked inflammation, worsening thrombocytopenia (platelets 98 to 11 ×10³/μL), coagulopathy (PT-INR 1.53 to 2.12), severe hypoalbuminemia (1.7 g/dL), and progressive renal dysfunction (creatinine 1.34 to 1.97 mg/dL), which preceded initiation of CHDF. PT: prothrombin time; INR: international normalised ratio; ICU: intensive care unit; CHDF: continuous hemodiafiltration; IF: International Federation of Clinical Chemistry and Laboratory Medicine (IFCC) method;

Parameter	Value on admission (ER)	Value on ICU day 7 (ICU admission)	Reference Range	Unit
Aspartate transferase	31	29	13-30	U/L
Alanine transaminase	19	22	10-42	U/L
Lactate dehydrogenase (IF)	212	412	124-222	U/L
Alkaline phosphatase (IF)	558	234	38-113	U/L
Creatine kinase	96	53	59-248	U/L
Amylase	38	62	44-132	U/L
Gamma-glutamyl transferase	102	53	13-64	U/L
Direct bilirubin	0.8	5.9	0-0.3	mg/dL
Total bilirubin	1.3	8.7	0.4-1.5	mg/dL
Blood urea nitrogen	37	88.1	8-20	mg/dL
Creatinine	1.34	1.97	0.65-1.07	mg/dL
Sodium	132	139	138-145	mEq/L
Potassium	3	4.9	3.6-4.8	mEq/L
Chloride	96	107	101-108	mEq/L
Calcium	7.5	7.7	8.8-10.1	mg/dL
Phosphate	2.3	4.2	2.7-4.6	mg/dL
Total protein	5.1	4.1	6.6-8.1	g/dL
Albumin	1.7	1.7	4.1-5.1	g/dL
Glucose	52	269	73-109	mg/dL
C-reactive protein	16.19	15.74	0-0.14	mg/dL
Procalcitonin	2.1		0-0.49	ng/mL
B‐type natriuretic peptide	49.2		≤ 18.4	pg/mL
White blood cells	13	31.6	3.3-8.6	x10^3^/μL
Red blood cell	2.93	2.6	4.35-5.55	x10^6^/μL
Hemoglobin	7.9	7.1	13.7-16.8	g/dL
Hematocrit	24.1	21.5	40.7-50.1	%
Mean corpuscular volume	82.3	82.7	83.6-98.2	fL
Platelet	98	11	158-348	x10^3^/μL
Prothrombin time	55	36	*≥*70	%
PT-INR	1.53	2.12	0.85–1.15	
Activated partial thromboplastin time	54.6	68.5	24-39	sec
Lactate	2.59	8.39	1.0–1.5	mmol/L

**Video 1:** Transthoracic echocardiography apical four-chamber view on hospital day 6, demonstrating preserved left ventricular systolic function In retrospect, the coronary sinus was also observed to be dilated, a finding consistent with a persistent left superior vena cava.

On hospital day 2, a central venous catheter was inserted into the right internal jugular vein for vasopressor administration. Because the patient developed progressive anasarca, thrombocytopenia, persistent fever, and worsening renal dysfunction, TAFRO (Thrombocytopenia, Anasarca, Fever, Reticulin fibrosis/Renal dysfunction, and Organomegaly) syndrome was suspected.

**Figure 3:**
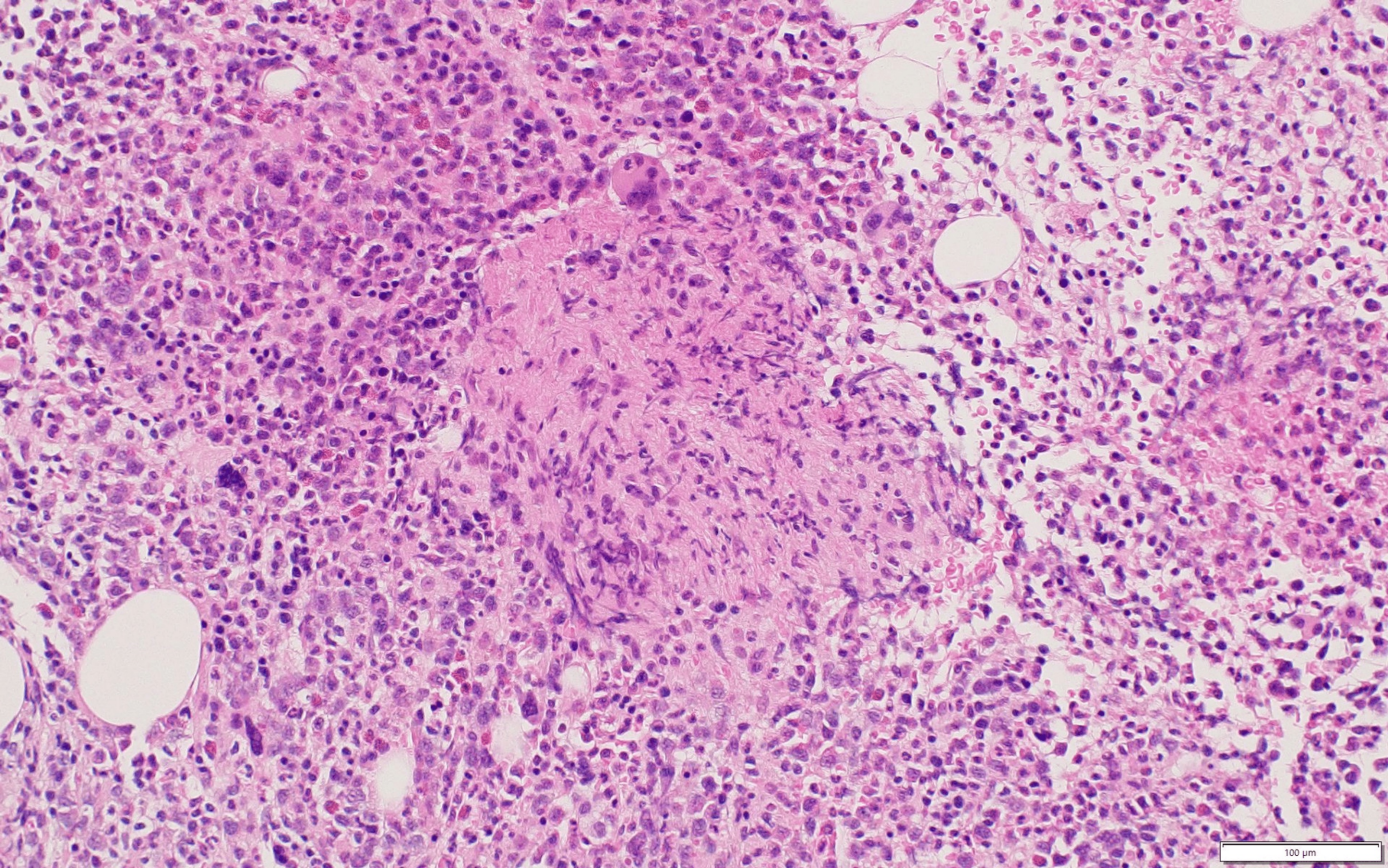
Hematoxylin and eosin-stained bone marrow showing increased megakaryocytes with background marrow fibrosis.

**Figure 4:**
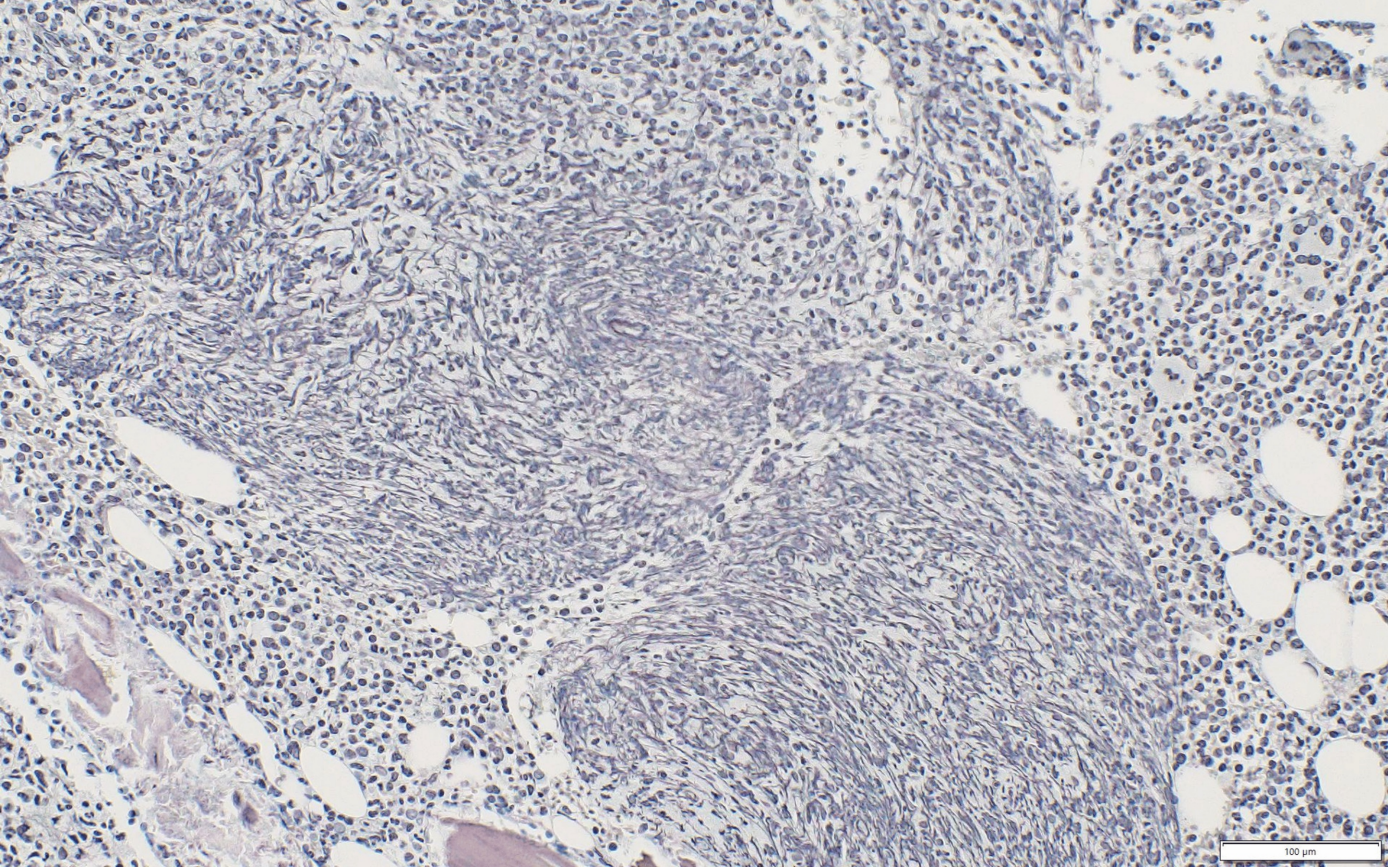
Reticulin-stained bone marrow Increased reticulin fibers are present (MF-1 to MF-2), consistent with reticulin fibrosis described in TAFRO syndrome in the appropriate clinical context. TAFRO: Thrombocytopenia, Anasarca, Fever, Reticulin fibrosis/Renal dysfunction, and Organomegaly

A random skin biopsy from the thigh and a bone marrow biopsy were performed on hospital day 6. The pathology reports, which became available after ICU admission, showed no malignant or clearly atypical cells in the skin, while the bone marrow demonstrated megakaryocytic hyperplasia with reticulin fibrosis (MF-1 to MF-2), a pattern considered consistent with TAFRO syndrome in the context of the patient’s clinical findings (Figures 3, 4).

On hospital day 7, the patient was transferred to the intensive care unit (ICU) due to respiratory failure requiring mechanical ventilation and acute kidney injury necessitating continuous hemodiafiltration (CHDF).

**Figure 5:**
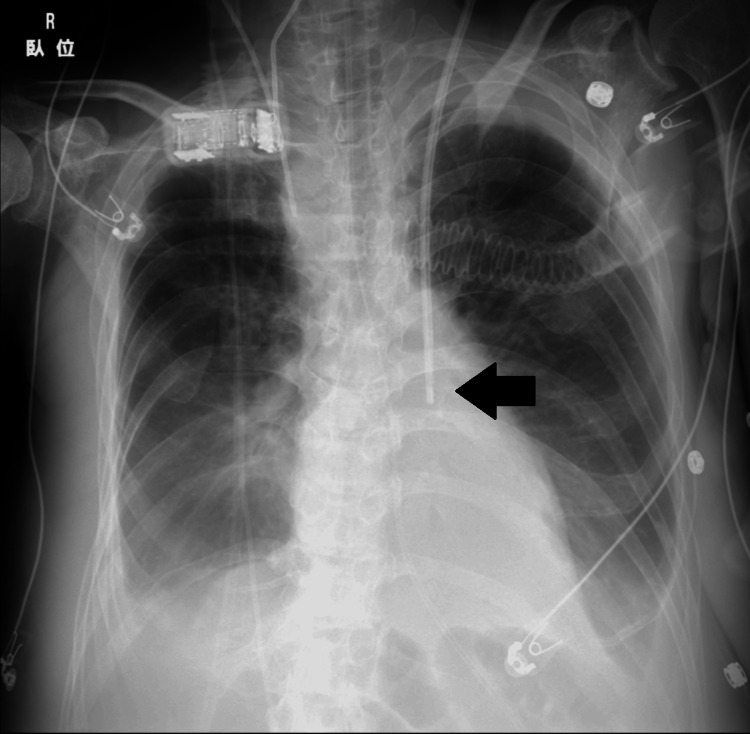
Chest radiograph after hemodialysis catheter insertion, showing the catheter descending along the left border of the mediastinum (arrow).

Because the right internal jugular vein was already occupied by a central venous line and both femoral veins carried a high infection risk due to biopsy sites, the left internal jugular vein was selected for hemodialysis catheter insertion. The catheter was inserted under ultrasound guidance and secured at a depth of 18 cm from the skin. After placement, a chest radiograph revealed an anomalous left-sided course of the catheter (Figure 5). A review of the initial CT scan confirmed the presence of a PLSVC, which was identified as Type IIIb according to Schummer's classification [3]. As previous case reports had documented successful hemodialysis via a PLSVC, CHDF was initiated using the same catheter.

**Figure 6:**
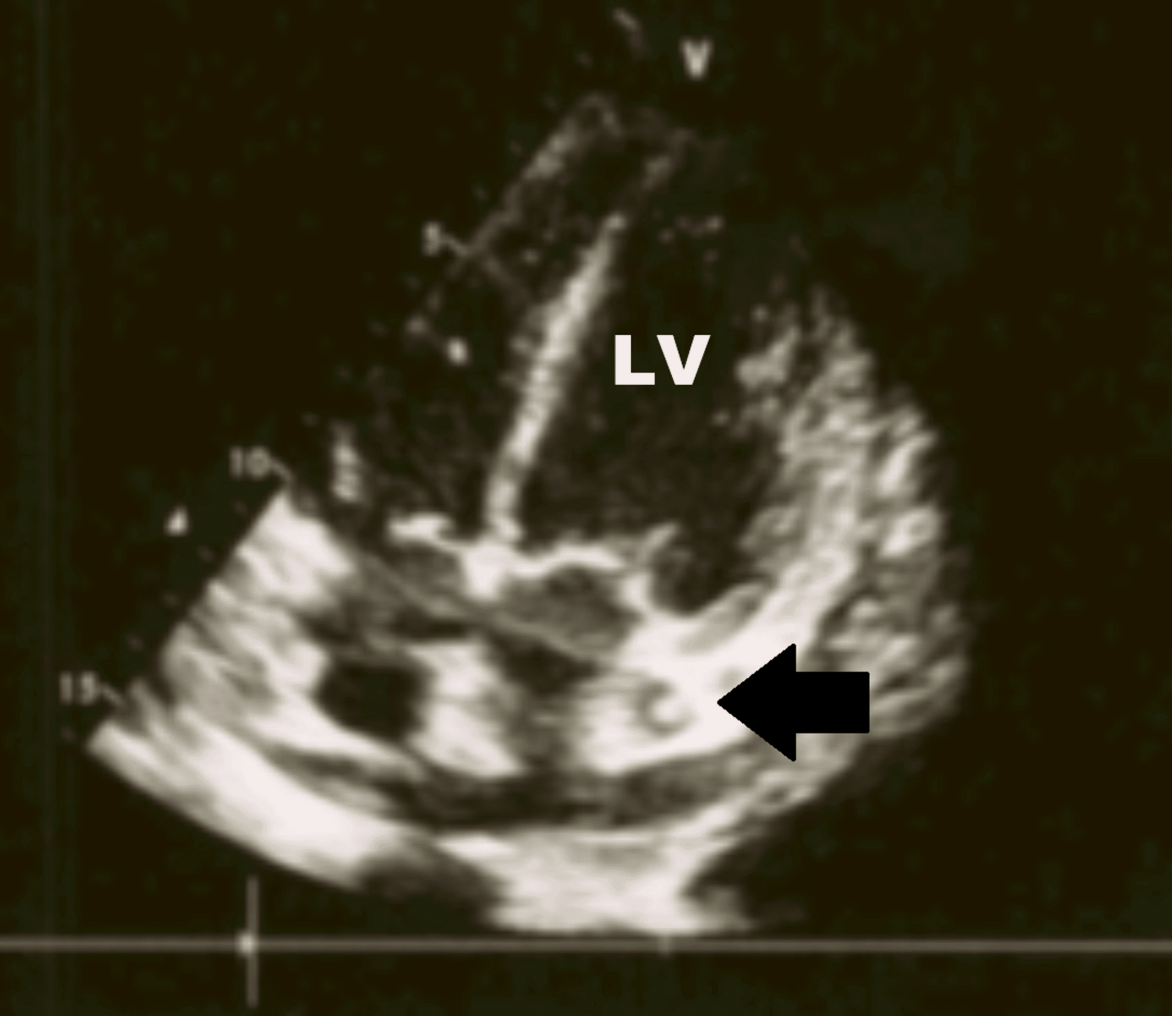
The hemodialysis catheter (arrow) is visualized as a hyperechoic structure within the PLSVC. LV: left ventricle; PLSVC: persistent left superior vena cava

**Figure 7:**
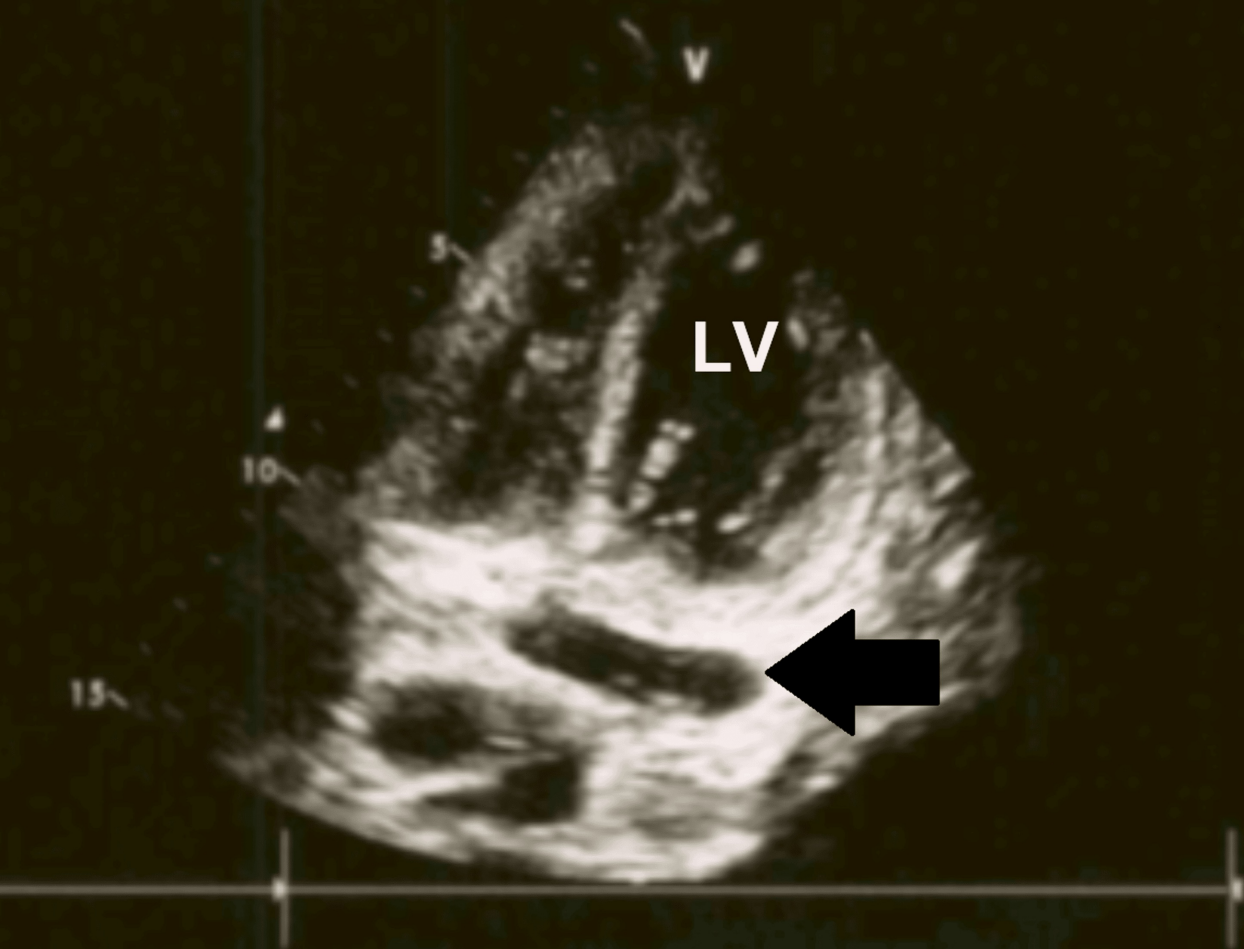
The coronary sinus (arrow) is visualized immediately caudal to the catheter tip. LV: left ventricle

On ICU day two (hospital day 8), routine bedside TTE revealed that the catheter tip was positioned near the curvature of the PLSVC, where it drained into the coronary sinus, a location associated with a risk of vascular perforation (Figures 6, 7).

Under real-time echocardiographic guidance, the catheter was withdrawn by 5 cm to a final depth of 13 cm at the skin, so that its tip was repositioned to a more cephalad segment of the PLSVC away from the high-risk curvature near the coronary sinus. This adjustment preserved adequate catheter function, maintaining blood flow for CHDF.

CHDF was continued for nine days without complications using the repositioned catheter. During this period, the patient also underwent three sessions of plasma exchange for TAFRO syndrome. Blood flow rates of 90-100 mL/minute were consistently achieved. His condition gradually improved, allowing extubation on ICU day 7. The hemodialysis catheter was removed 14 days after insertion, following recovery of renal function.

## Discussion

This case demonstrates the utility of bedside TTE for managing central venous access in patients with PLSVC, particularly when fluoroscopy is unavailable. Our patient required CHDF for acute kidney injury complicated by septic shock and TAFRO syndrome, which is a systemic inflammatory disorder of unknown etiology [4,5].

These features restricted our vascular access options. The femoral route was avoided due to infection risks related to recent biopsy sites, while the right internal jugular vein was already occupied. Consequently, the left internal jugular vein was selected, leading to incidental cannulation of a PLSVC.

Subclavian access was considered; however, thrombocytopenia/coagulopathy and the limited ability to compress the subclavian site made an additional puncture less favorable, and the team preferred to continue using the already functioning catheter after CHDF had been initiated. In retrospect, if PLSVC had been recognized before cannulation, a right-sided approach (including right subclavian access) could have been considered. Several reports have described transvenous devices being inserted via a PLSVC, including central venous catheters and cardiac implantable electronic devices [6-8]. Although rare, the use of a PLSVC for hemodialysis catheterization has also been reported as a viable alternative when conventional access is limited [3].

For central venous catheters placed in the normal right SVC, bedside ultrasound techniques, such as direct visualization of the catheter tip within the right atrium or detection of agitated saline microbubbles, have been evaluated as accurate and faster alternatives to chest radiography for confirming tip position [9]. In contrast, fluoroscopic guidance is generally recommended for confirming catheter position in patients with PLSVC [3].

**Figure 8:**
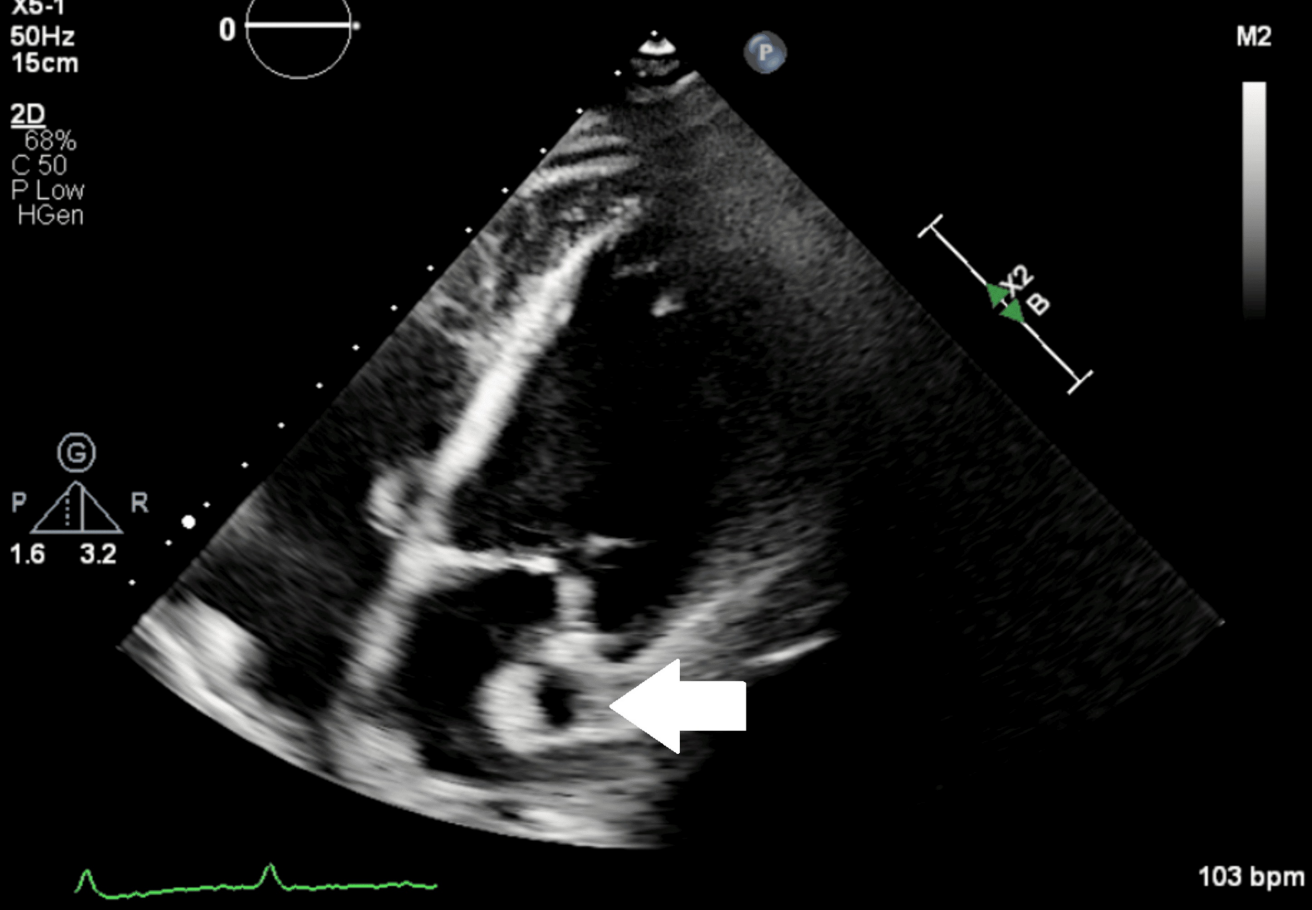
Transthoracic echocardiography performed in the clinical laboratory after admission shows the persistent left superior vena cava (arrow) in an apical four-chamber view. The heart serves as an acoustic window, allowing the anomalous vein to be visualized adjacent to the left atrium.

**Figure 9:**
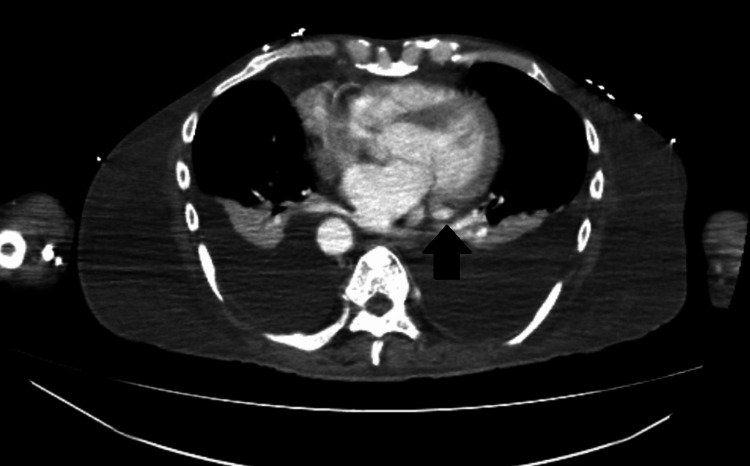
Contrast-enhanced chest CT shows the persistent left superior vena cava (arrow) running along the left side of the mediastinum in close relationship to the heart. This anatomical configuration explains why the vessel can be depicted in the apical four-chamber echocardiographic view.

However, transferring hemodynamically unstable patients to the radiology suite entails significant risk. In this case, bedside TTE allowed clear visualization of the PLSVC and the catheter tip, enabling real-time adjustment without patient transport. Previous reports have also described the incidental use of TTE to identify PLSVC after catheterization [10], supporting its utility as a non-invasive bedside imaging tool. When an apical four-chamber view is obtained with TTE, the PLSVC can be relatively easily visualized using the heart as an acoustic window (Figure 8). The corresponding chest CT (Figure 9) illustrates the anatomical relationship between the PLSVC and the heart, which explains why the anomalous vein can be captured in this echocardiographic view. Thus, TTE represents a practical and safe alternative to fluoroscopy, particularly for ICU patients.

In addition to post-placement confirmation, bedside echocardiography may also support safer left-sided catheter insertion. When feasible, briefly assessing the right atrium and the SVC region during guidewire advancement may help confirm an expected trajectory and reduce inadvertent cannulation of unintended vessels. If the guidewire cannot be visualized in the SVC or right atrium at an appropriate depth, the operator should pause further advancement and consider abnormal venous anatomy, including PLSVC, and reassess with additional imaging. In the present case, TTE was not used during cannulation, but it enabled bedside risk assessment and immediate repositioning once the deep tip position near the coronary sinus was recognized.

Key modalities used to assess catheter trajectory and tip position after left-sided cannulation are summarized in Table 2.

**Table 2:** Key modalities to confirm catheter trajectory and tip position after left-sided cannulation CXR: chest radiography; ECG: electrocardiography; TTE: transthoracic echocardiography; TEE: transesophageal echocardiography; CT: computed tomography; SVC: superior vena cava; RA: right atrium; CAJ: cavoatrial junction; PLSVC: persistent left superior vena cava

Modality	Advantages	Key checkpoints/landmarks	Limitations
CXR	Widely available; rapid bedside screening	Catheter crosses the midline via the left brachiocephalic vein; tip projects to the lower SVC/CAJ region (around the carina level).	Limited for exact tip depth; projection dependent.
Intracavitary ECG	Real-time; no radiation; estimates CAJ	P-wave amplitude increases as the tip approaches the CAJ; atypical P-wave patterns (e.g., negative P in lead II) may suggest PLSVC [11].	Requires sinus rhythm; limited in paced rhythm/arrhythmias.
TTE	Non-invasive; portable; bedside adjustment	Subcostal bicaval or four-chamber view: visualize the guidewire/tip in the RA/SVC as a hyperechoic line; saline flush from the catheter: confirm microbubbles in the RA.	Window dependent; operator dependent.
TEE	Higher resolution when TTE windows are limited	Mid-esophageal bicaval view: confirm the wire/tip entering the RA from the SVC.	Invasive; requires sedation/expertise.
Fluoroscopy	Reference standard: real-time trajectory assessment	Contrast delineates the venous pathway and tip position.	Radiation/contrast; often requires transport.
CT	Definitive anatomic delineation; evaluates associated anomalies/complications	Contrast CT provides a three-dimensional assessment of venous anatomy and tip position.	Transport/radiation (and contrast, if used).

Another critical consideration is the final catheter tip position. Although the PLSVC can be used as an alternative route for vascular access, deep insertion near the coronary sinus carries a risk of severe complications, including pericardial effusion and mediastinal hematoma [12,13]. These events are thought to result from mechanical irritation or perforation where the PLSVC curves toward the coronary sinus. In our case, the catheter tip was initially located at this high-risk curvature. After withdrawing it several centimeters under real-time TTE guidance, no complications occurred, and dialysis was performed successfully. This suggests that maintaining a relatively shallow catheter position, cephalad to the coronary sinus, may be the key to safe hemodialysis via a PLSVC. For non-dialysis central venous catheters in patients with normal venous anatomy, when a catheter is inserted from the right side (e.g., the right internal jugular or right subclavian vein), It is generally recommended to place the catheter tip in "zone B" of the SVC, which is within the SVC but above the level of the carina and proximal to the pericardial reflection, to reduce the risk of cardiac tamponade in the event of perforation [14]. For left-sided approaches, a “necessary compromise” in which the tip is advanced into “zone A” in the lower SVC has been proposed so that the catheter runs parallel to the vessel wall rather than abutting the right lateral wall perpendicularly. In contrast, the Japanese Society for Dialysis Therapy guidelines state that the tip of a hemodialysis catheter should preferably float freely within the right atrium, and that tips positioned in the SVC or innominate vein may later develop inadequate inflow or outflow if the side holes contact the vessel wall [15]. In a patient with a type IIIb PLSVC according to Schummer’s classification, advancing a hemodialysis catheter through the coronary sinus into the right atrium would require traversing a sharply curved, thin-walled segment and is therefore considered to carry an unacceptably high risk of perforation. Our strategy of maintaining the catheter tip in a more proximal segment of the PLSVC, away from the coronary sinus, appears reasonable in this anatomical context.

From a clinical standpoint, when a catheter inserted from the left internal jugular vein follows an unusual course on chest radiography, the presence of a PLSVC should be suspected. However, an aberrant left-sided catheter course has a differential diagnosis, including inadvertent arterial cannulation (e.g., the descending thoracic aorta), malposition into small thoracic veins (e.g., the left internal thoracic, left superior intercostal, or pericardiophrenic vein), or extravascular placement; therefore, confirmation of the venous pathway and tip position with additional imaging is warranted when the course is atypical [16]. In such cases, bedside TTE can rapidly determine the venous anatomy and confirm the tip position. This approach minimizes the risk of vascular injury and avoids the hazards of patient transport, which is particularly valuable in critically ill patients. Routine awareness of this anatomical variant and familiarity with echocardiographic assessment can therefore improve procedural safety in the ICU.

## Conclusions

This case demonstrates that bedside TTE can be effectively used to confirm and adjust the position of a hemodialysis catheter placed within a PLSVC. Two key lessons emerge from this case. First, TTE offers a practical, non-invasive method for bedside catheter repositioning in critically ill patients, which may reduce the need for fluoroscopic guidance when patient transport is hazardous. Second, placing the catheter tip at a relatively shallow position above the coronary sinus may help prevent potentially life-threatening mechanical complications while preserving adequate dialysis performance.
